# Improved Single Inertial-Sensor-Based Attitude Estimation during Walking Using Velocity-Aided Observation

**DOI:** 10.3390/s21103428

**Published:** 2021-05-14

**Authors:** Duc Cong Dang, Young Soo Suh

**Affiliations:** Department of Electrical Engineering, Electronic and Computer Engineering, University of Ulsan, Ulsan 44610, Korea; congdd.ac@gmail.com

**Keywords:** inertial sensor, body-mounted, attitude estimation, Kalman filter, velocity observation

## Abstract

This paper presents a Kalman filter-based attitude estimation algorithm using a single body-mounted inertial sensor consisting of a triaxial accelerometer and triaxial gyroscope. The proposed algorithm has been developed for attitude estimation during dynamic conditions such as walking and running. Based on the repetitive properties of the velocity signal of human gait during walking, a novel velocity-aided observation is used as a measurement update for the filter. The performance has been evaluated in comparison to two standard Kalman filters with different measurement update methods and a smoother algorithm which is formulated in the form of a quadratic optimization problem. Whereas two standard Kalman filters give maximum 5 degrees in both pitch and roll error for short walking case, their performance gradually decrease with longer walking distance. The proposed algorithm shows the error of about 3 degrees in 15 m walking case, and indicate the robustness of the method with the same performance in 75 m trials. As far as the accuracy of the estimation is concerned, the proposed method achieves advantageous results due to its periodic error correction capability in both short and long walking cases at varying speeds. In addition, in terms of practicality and stability, with simple parameter settings and without the need of all-time data, the algorithm can achieve smoothing-algorithm-performance level with lower computational resources.

## 1. Introduction

The advent of Micro Electro Mechanical Systems (MEMS) technology has made it possible for Inertial Measurement Units (IMU) to attract significant attention due to their miniaturized, low power consumption, and low cost [1]. In particular, the use of inertial sensors has been playing a key role in a wide range of applications requiring attitude (i.e., roll and pitch) and/or heading (i.e., yaw) information such as indoor localization [2,3], unmanned underwater vehicles (UAVs) and spacecraft inertial navigation [4,5], human activity and gesture recognition [6,7,8,9], robots and drones control and stabilization [10,11].

An IMU, typically composed of a three-axis accelerometer, and a three-axis gyroscope, could observe the linear and angular motions, of the target in three-dimensional space to determine attitude in relation to the inertial system. In fact, by combining the inertial sensors with a magnetometer, forming an attitude and heading reference system (AHRS) [12,13,14], much of the current work on the inertial sensing-based orientation estimation has centered on 3-D orientations. However, only the attitude is required without the heading information for various applications such as robot and vehicle stability control [15], human balance issues [16], and gait analysis [17,18,19]. In this paper, the magnetometer is omitted and only the attitude estimation problem is considered using a six-DOF inertial sensor comprised of a triaxial accelerometer and a triaxial gyroscope.

The inertial sensing-based attitude estimation problem usually relies on the following concepts. An accelerometer determines the orientation by sensing the gravitational acceleration about its axes during static conditions. A gyroscope measures the angular rate of rotation along its axes and integrating these rates over time, results in the orientation angles. However, the integration process also accumulates any gyroscope bias or measurement error, and cause attitude drift errors. During dynamic conditions, the accelerometer measures external acceleration as well as gravitational acceleration. Thus the attitude estimation based on the corrupted acceleration is not accurate. The classic strategy to deal with this issue is to use a threshold-based switching technique, which makes use of the fact that the accelerometer should measure only gravitational acceleration (norm 9.81 m/s${}^{2}$) during static conditions [20]. When the norm of the accelerometer output exceeds the gravitational acceleration norm by some pre-defined threshold, it can be determined that there is external acceleration (that is, dynamic conditions). The estimation then switches the measurement signal to gyroscope only until the threshold condition is not met. This method is simple but will be affected by incorrect dynamic state detection and prolonged dynamic conditions. Therefore, a proper fusion of the accelerometer and gyroscope signals is essential in order to address the external acceleration problem and obtain high accuracy attitude estimation.

Recent gait analysis studies show the increasing use of wearable inertial sensor instead of camera-based laboratory systems for measuring attitude during gait [21,22,23]. However, most of the works focus on analysis by monitoring the angle of various joints on the lower extremities (e.g., foot, shank, calf or thigh) whereas only a small number of studies have analyzed upper limb function despite the fact that the upper limbs play an important role in balance and stability maintenance, reduction of mechanical loads on tissue, and improved gait efficiency [24,25,26]. In this article, we focus on estimating attitude of human body during locomotion, particularly upper limb during walking using single IMU.

During walking, the accelerometer outputs are constantly corrupted by linear acceleration caused by the movement of the body, which degrades the attitude estimation performance. For a short distance walking, attitude estimation can be estimated by just integrating gyroscope outputs without using corrupted accelerometer data. However, the attitude estimation becomes inaccurate for longer distance walking.

The key contribution of this paper is to provide a new measurement updating approach to the issue of attitude estimation in human walking scenarios by using some constraints obtained from the almost periodic nature of walking. Experiments are carried out to evaluate the accuracy of the proposed algorithm in attitude estimation with different sensor attachment positions. The properties of the proposed approach are discussed in comparison with two standard Kalman filters with different measurement updating methods and a smoother algorithm using quadratic optimization technique.

The remaining sections are organized as follows: In Section 2, basic definitions and equations of a quaternion-based Kalman filter is given. Measurement updating methods are illustrated in Section 3 with a typical two level measurement update and a proposed method, where a repetitive property of walking velocity signal is used. Experimental setup and results are indicated in Section 4. Discussion, limitations and future works are given in Section 5.

## 2. Standard Indirect Kalman Filter for Attitude Estimation

The coordinate transformation of a 3×1 position vector *r* between the body attached sensor coordinate frame and the fixed North-East-Down world coordinate frame is expressed as follows:

$$r_{b}=C_{w}^{b}r_{w},$$

where the right subscripts *b* and *w* imply the corresponding vectors are expressed in the body and world coordinate frames, and $C_{w}^{b}\in SO\left(3\right)$, simply denoted as *C*, is the rotation matrix that represents the orientation of the world frame with respect to the body frame. It contains the three unit column vectors of the world coordinate expressed in body coordinate, and can either be represented by the Euler angles or the quaternion. In this paper, quaternion representation is chosen due to its efficiency in computation. The relationship between quaternion *q* and its corresponding rotation matrix C(q) is given by

$$C\left(q\right)=\left[\begin{array}{ccc} 2q_{0}^{2}+2q_{1}^{2}-1 & 2q_{1}q_{2}+2q_{0}q_{3} & 2q_{1}q_{3}-2q_{0}q_{2}\\ 2q_{1}q_{2}-2q_{0}q_{3} & 2q_{0}^{2}+2q_{2}^{2}-1 & 2q_{2}q_{3}+2q_{0}q_{1}\\ 2q_{1}q_{3}+2q_{0}q_{2} & 2q_{2}q_{3}-2q_{0}q_{1} & 2q_{0}^{2}+2q_{3}^{2}-1 \end{array}\right].$$

In this paper, quaternion *q* is estimated using standard Kalman filter during walking, where the initial heading is arbitrarily chosen.

The kinematic equation of a rotation is given by [12]

(1) $$\dot{q}=\frac{1}{2}q⊗\left[\begin{array}{c} 0\\ \omega \end{array}\right]=\frac{1}{2}\Omega \left(\omega \right)q,$$

where $\omega \in R^{3}$ is the body angular velocity, operation ⊗ represents quaternion multiplication and the symbol Ω(ω) is defined by

$$\Omega \left(\omega \right)=\left[\begin{array}{cc} 0 & \omega^{\prime }\\ \omega & -[\omega \times ] \end{array}\right],$$

where [ω×] is the cross product operation on vector ω, and $\omega^{\prime }$ is the transpose of ω.

Accelerometer and gyroscope outputs $y_{a}\in R^{3}$ and $y_{g}\in R^{3}$ are modeled as follows:

(2) $$\begin{array}{cc} y_{a} & =C\left(q\right)\tilde{g}+a_{b}+n_{a},\\ y_{g} & =\omega +b_{g}+n_{g}. \end{array}$$

where $a_{b}\in R^{3}$ is unknown external acceleration and $b_{g}\in R^{3}$ is gyroscope bias. The local gravitation vector $\tilde{g}\in R^{3}$ is given by

$$\tilde{g}≜\left[\begin{array}{c} 0\\ 0\\ g \end{array}\right],$$

where g∈R is the magnitude of the gravitation and $n_{a}\in R^{3},n_{g}\in R^{3}$ are sensor noises. It is assumed that the sensor noises are zero-mean white Gaussian and their covariances are given by

$$E\left\{n_{a}\left(t\right)n_{a}^{\prime }\left(s\right)\right\}=r_{a}I_{3}\delta \left(t-s\right),E\left\{n_{g}\left(t\right)n_{g}^{\prime }\left(s\right)\right\}=r_{g}I_{3}\delta \left(t-s\right),$$

where $r_{a}\in R$ and $r_{g}\in R$ are positive scalars.

Let ${\hat{b}}_{g}\in R^{3}$ be an estimate of the gyroscope bias $b_{g}$ with an error $b_{g,e}$

(3) $$b_{g}={\hat{b}}_{g}+b_{g,e}.$$

The gyroscope bias $b_{g}$ can be modeled as nearly constant

(4) $${\dot{b}}_{g}=n_{b_{g}},$$

where a small zero-mean Gaussian process noise $n_{b_{g}}$ is added with covariance $Q_{b_{g}}\delta \left(t-s\right)=E\left\{n_{b_{g}}\left(t\right)n_{b_{g}}^{\prime }\left(s\right)\right\}$ so that the bias estimation is not stopped soon.

Let $\hat{q}\in R^{4}$ be an estimate of *q* computed from bias-corrected gyroscope output by

(5) $$\dot{\hat{q}}=\frac{1}{2}\hat{q}⊗\left[\begin{array}{c} 0\\ y_{g}-{\hat{b}}_{g} \end{array}\right].$$

Since $y_{g}-{\hat{b}}_{g}\neq \omega$ due to sensor noise and bias estimation error, the estimated quaternion $\hat{q}$ is not the same as the true value *q*. The estimation error ${\bar{q}}_{e}\in R^{4}$ can be represented in the multiplicative form

(6) $$q={\bar{q}}_{e}⊗\hat{q},$$

or in the rotation matrix expression

(7) $$C\left(q\right)=C\left(\hat{q}\right)C\left({\bar{q}}_{e}\right).$$

Assuming that attitude error is small, we can approximate ${\bar{q}}_{e}\in R^{4}$ by 3×1 vector $q_{e}$ as follows:

(8) $${\bar{q}}_{e}\approx \left[\begin{array}{c} 1\\ q_{e} \end{array}\right]\in \left[\begin{array}{c} R\\ R^{3} \end{array}\right],$$

and we can approximate $C({\bar{q}}_{e})$ as follows [12]:

(9) $$C\left({\bar{q}}_{e}\right)\approx \left[\begin{array}{ccc} 1 & 2q_{e,3} & -2q_{e,2}\\ -2q_{e,3} & 1 & 2q_{e,1}\\ 2q_{e,2} & -2q_{e,1} & 1 \end{array}\right]=I-2\left[q_{e}\times \right].$$

The state of an indirect Kalman filter is defined by

(10) $$x≜\left[\begin{array}{c} q_{e}\\ b_{g,e} \end{array}\right]\in R^{6}.$$

We have the following state equation for an indirect Kalman filter:

(11) $$\dot{x}\left(t\right)=\left[\begin{array}{c} {\dot{q}}_{e}\\ {\dot{b}}_{g,e} \end{array}\right]=Ax\left(t\right)+\left[\begin{array}{c} -\frac{1}{2}C{\left(\hat{q}\right)}^{\prime }n_{g}\\ n_{b_{g}} \end{array}\right],$$

where

$$A=\left[\begin{array}{cc} 0_{3\times 3} & -\frac{1}{2}C{\left(\hat{q}\right)}^{\prime }\\ 0_{3\times 3} & 0_{3\times 3} \end{array}\right].$$

Covariance of the process noise is as follows:

$$\begin{array}{cc} E\left\{\left[\begin{array}{c} -\frac{1}{2}C{\left(\hat{q}\right)}^{\prime }n_{g}\left(t\right)\\ n_{b_{g}\left(t\right)} \end{array}\right]\right. & \left.{\left[\begin{array}{c} -\frac{1}{2}C{\left(\hat{q}\right)}^{\prime }n_{g}\left(s\right)\\ n_{b_{g}}\left(s\right) \end{array}\right]}^{\prime }\right\}\\ \; & =\delta \left(t-s\right)\left[\begin{array}{cc} \frac{1}{4}C\left(\hat{q}\right)r_{g}I_{3}C{\left(\hat{q}\right)}^{\prime } & 0\\ 0 & Q_{b_{g}} \end{array}\right]. \end{array}$$

The measurement equation for an indirect Kalman filter is derived from the sensor output equations. Inserting (7) and (9) into (2), we have

(12) $$y_{a}-C\left(\hat{q}\right)\tilde{g}=2C\left(\hat{q}\right)\left[\tilde{g}\times \right]q_{e}+a_{b}+n_{a}.$$

(13) $$y_{g}-{\hat{b}}_{g}=b_{g,e}+\omega +n_{g}.$$

Equations (11)–(13) constitute a continuous standard indirect Kalman filter for attitude estimation problem.

## 3. Proposed Measurement Updating Methods

In this section, a typical measurement updating method using two level measurement update is given. The algorithm based on this technique together with a new velocity-aided observation is proposed in the later of this section.

### 3.1. Two Level Measurement Updates

When the target is being transformed, including moving and rotating, only the state update Equation (11) is used in the filter without any measurement update. The integrating process might cause accumulate error if the error and bias are not compensated.

A so-called zero-velocity update (ZUPT) technique uses the fact that the velocity must be zero whereas the object is not moving in a specific time interval to reduce the error drift in the integration part of a standard Kalman filter, including position, velocity, and orientation estimations. This technique is usually used with foot-mounted inertial navigation systems, where the foot is stationary during stance phases. However, with the sensor unit attached on human upper body rather than on the feet, it is relatively difficult since there is no periodical instance of zero-velocity during walking.

A more appropriate approach for upper body attitude estimation is to apply gravity measurement (GM) model and the zero-angular-rate update (ZARU) model at detected events. The gravity measurement model employs the acceleration measurement to compensate roll and pitch under the condition that no significant linear acceleration is present. The measured acceleration is considered to be due to gravitational acceleration only

(14) $$y_{a}-C\left(\hat{q}\right)\tilde{g}=2C\left(\hat{q}\right)\left[\tilde{g}\times \right]q_{e}+n_{a}.$$

Thus, the corresponding measurement equation in the prediction step is given as follows:

(15) $$\begin{array}{cc} K_{a,k} & =P_{k}^{-}H_{a,k}^{\prime }{\left(H_{a,k}P_{k}^{-}H_{a,k}^{\prime }+R_{a}\right)}^{-1}\\ P_{k} & =\left(I-K_{a,k}H_{a,k}\right)P_{k}{\left(I-K_{a,k}H_{a,k}\right)}^{\prime } \end{array}$$

(16) $${\hat{x}}_{k}={\hat{x}}_{k}^{-}+K_{a,k}\left(z_{a,k}-H_{a,k}{\hat{x}}_{k}\right),$$

where

(17) $$\begin{array}{cc} H_{a,k} & =\left[\begin{array}{cc} 2C\left({\hat{q}}_{k}\right)\left[\tilde{g}\times \right] & 0_{3\times 3} \end{array}\right]\\ z_{a,k} & =y_{a,k}-C\left({\hat{q}}_{k}\right)\tilde{g}. \end{array}$$

The ZARU model relies on the condition that there is no rotational motion at the detected ZARU events, and the gyroscope measurement is due to sensor bias only

(18) $$y_{g}-{\hat{b}}_{g}=b_{g,e}+n_{g}.$$

The measurement equation in the prediction step is given as follows:

(19) $$\begin{array}{cc} K_{g,k} & =P_{k}^{-}H_{g,k}^{\prime }{\left(H_{g,k}P_{k}^{-}H_{g,k}^{\prime }+R_{g}\right)}^{-1}\\ P_{k} & =\left(I-K_{g,k}H_{g,k}\right)P_{k}{\left(I-K_{g,k}H_{g,k}\right)}^{\prime } \end{array}$$

(20) $${\hat{x}}_{k}={\hat{x}}_{k}^{-}+K_{g,k}\left(z_{g,k}-H_{g,k}{\hat{x}}_{k}\right),$$

where

(21) $$\begin{array}{cc} H_{g,k} & =\left[\begin{array}{cc} 0_{3\times 3} & I_{3\times 3} \end{array}\right]\\ z_{g,k} & =y_{g,k}-{\hat{b}}_{g}. \end{array}$$

### 3.2. Velocity-Aided Observation

A standard Kalman filter based attitude estimation solution is presented in Section 2. To overcome the external acceleration problem during walking, we derive a new measurement equation, which uses the almost periodic property of human gait during walking. The derivation of this measurement equation is the main contribution of this paper.

**Figure 1:**
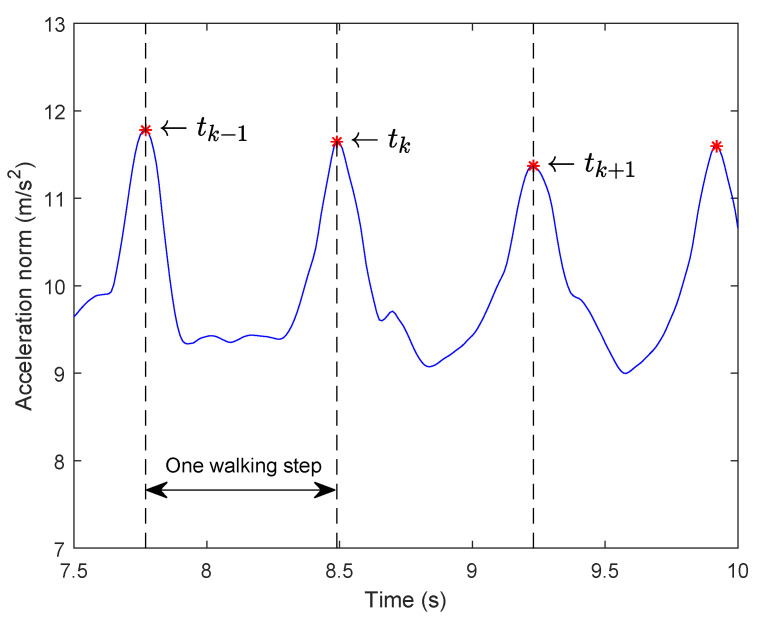
Acceleration norm signal and detected high peaks of a waist-mounted IMU during walking.

Figure 1 shows the acceleration norm of a waist-mounted IMU during walking, where we can observe the almost periodic pattern. In Figure 1, the denoted $t_{k}$ represents one peak time index, which corresponds to a double stance (DS) instant and $\left[t_{k-1},t_{k}\right]$ interval defines one walking step.

Velocities in the world coordinate system at $t_{1}$ and $t_{2}$ are related by the following equation:

(22) $$\begin{array}{cc} v_{t_{2}} & =v_{t_{1}}+\int_{t_{1}}^{t_{2}}C_{b}^{w}\left(t\right)a_{b}dt. \end{array}$$

Rewriting the velocity equation in the quaternion form including measurement noise term, we obtain

(23) $$\begin{array}{cc} v_{t_{2}} & =v_{t_{1}}+\int_{t_{1}}^{t_{2}}C{\left(q\right)}^{\prime }a_{b}dt\\ \; & =v_{t_{1}}+\int_{t_{1}}^{t_{2}}C{\left(q\right)}^{\prime }\left(y_{a}-C\left(q\right)\tilde{g}-n_{a}\right)dt\\ \; & =v_{t_{1}}-\tilde{g}\left(k_{2}-k_{1}\right)T+\int_{t_{1}}^{t_{2}}C{\left(q\right)}^{\prime }y_{a}dt-\int_{t_{1}}^{t_{2}}C{\left(q\right)}^{\prime }n_{a}dt. \end{array}$$

where

$$\begin{array}{cc} \int_{t_{1}}^{t_{2}}C{\left(q\right)}^{\prime }y_{a}dt & =\int_{t_{1}}^{t_{2}}{\left(I-2\left[q_{e}\times \right]\right)}^{\prime }C{\left(\hat{q}\right)}^{\prime }y_{a}dt\\ \; & =\int_{t_{1}}^{t_{2}}C{\left(\hat{q}\right)}^{\prime }y_{a}dt+2\left[q_{e}\times \right]\int_{t_{1}}^{t_{2}}C{\left(\hat{q}\right)}^{\prime }y_{a}dt\\ \; & =\int_{t_{1}}^{t_{2}}C{\left(\hat{q}\right)}^{\prime }y_{a}dt-2\left[\left(\int_{t_{1}}^{t_{2}}C{\left(\hat{q}\right)}^{\prime }y_{a}dt\right)\times \right]q_{e}. \end{array}$$

Rearranging (23), we obtain the following:

(24) $$\begin{array}{cc} \tilde{g}\left(k_{2}-k_{1}\right)T-\int_{t_{1}}^{t_{2}}C{\left(\hat{q}\right)}^{\prime }y_{a}dt=-2\left[\left(\int_{t_{1}}^{t_{2}}C{\left(\hat{q}\right)}^{\prime }y_{a}dt\right)\times \right]q_{e} & \\ -{\bar{n}}_{v,diff}-\int_{t_{1}}^{t_{2}}C{\left(q\right)}^{\prime }n_{a}dt & . \end{array}$$

where ${\bar{n}}_{v,diff}$ is defined by

$${\bar{n}}_{v,diff}=v_{t_{2}}-v_{t_{1}}.$$

If we know ${\bar{n}}_{v,diff}$, (24) can be considered as an attitude measurement equation estimating $q_{e}$.

We have done a regular straight walking experiment with normal person without any movement disorder. The participants walk along a straight corridor from standing still in their preferred speed and in a most comfortable way. We found from the experiment that people tend to maintain their walking pace at the same speed and the velocity difference at double stance (DS) instant of two consecutive strides is relatively small. In this case, ${\bar{n}}_{v,diff}\approx 0$ and (24) can be used as a measurement equation even if we do not know the walking speed $v_{t_{1}}$ and $v_{t_{2}}$. The velocity-aided measurement equation can be formed as follows:

$$\begin{array}{cc} K_{v,k} & =P_{k}^{-}H_{v,k}^{\prime }{\left(H_{v,k}P_{k}^{-}H_{v,k}^{\prime }+R_{v}\right)}^{-1}\\ {\hat{x}}_{k} & ={\hat{x}}_{k}^{-}+K_{v,k}\left(z_{v,k}-H_{v,k}{\hat{x}}_{k}\right)\\ P_{k} & =\left(I-K_{v,k}H_{v,k}\right)P_{k}{\left(I-K_{v,k}H_{v,k}\right)}^{\prime } \end{array}$$

where

$$\begin{array}{cc} H_{v,k} & =\left[\begin{array}{cc} -2\left[\left(\sum_{i=k-1}^{k}C{\left({\hat{q}}_{i}\right)}^{\prime }y_{a,i}\right)\times \right] & 0_{3\times 3} \end{array}\right]\\ z_{v,k} & =\tilde{g}\left(t_{k}-t_{k-1}\right)-\sum_{i=k-1}^{k}C{\left({\hat{q}}_{i}\right)}^{\prime }y_{a,i}. \end{array}$$

Since $n_{a}$ is assumed to be white Gaussian noise, the term ${\bar{n}}_{v}=\int_{t_{1}}^{t_{2}}C{\left(q\right)}^{\prime }n_{a}dt$ can also be treated as white Gaussian noise

$$\begin{array}{cc} R_{v}=E\left\{{\bar{n}}_{v}{\bar{n}}_{v}^{\prime }\right\} & =\int_{t_{1}}^{t_{2}}\int_{t_{1}}^{t_{2}}C{\left(q\right)}^{\prime }E\left\{n_{a}n_{a}^{\prime }\right\}C\left(q\right)dt\\ \; & =\left(t_{2}-t_{1}\right)r_{a}I_{3}=\left(k_{2}-k_{1}\right)Tr_{a}I_{3}. \end{array}$$

The remaining question is when we can apply (24) for the filter’s measurement updating. In other words, we need a condition to determine whether the term ${\bar{n}}_{v,diff}$ is small enough.

Inserting (2) into (22), we obtain (noise terms are ignored)

$$v_{t_{2}}\approx v_{t_{1}}+\int_{t_{1}}^{t_{2}}\left(C_{b}^{w}\left(t\right)y_{a}-\tilde{g}\right)dt.$$

Noting that $\tilde{g}$ is a constant, we obtain

(25) $$v_{t_{2}}\approx v_{t_{1}}-\tilde{g}\left(t_{2}-t_{1}\right)+\int_{t_{1}}^{t_{2}}C_{b}^{w}\left(t\right)y_{a}dt.$$

Now we factorize $C_{b}^{w}\left(t\right)$ into two rotation matrices

(26) $$C_{b}^{w}\left(t\right)=C_{bt_{1}}^{w}C_{b}^{bt_{1}}\left(t\right).$$

The rotation matrix $C_{bt_{1}}^{w}$ denotes the attitude of the body frame at the time $t_{1}$ with respect to the world coordinate frame, whereas $C_{b}^{bt_{1}}\left(t\right)$ is calculated using the current attitude estimation at the time *t* relative to the last pose $t_{1}$. The later only requires the integration of rotation rate from time $t_{1}$ to time *t* with the initial condition for the rotation matrix $C_{b}^{bt_{1}}$ at the start of the integration period is $C_{bt_{1}}^{bt_{1}}$, which is the identity matrix. Thus it can be pre-integrated in the body frame of pose $t_{1}$ without the actual attitude of the target at the time $t_{1}$. This concept had been presented in [27], where the inertial observations are calculated from pose to pose in the body coordinate frame other than in world coordinate frame.

Inserting (26) into (25), we obtain

(27) $${\bar{n}}_{v,diff}=v_{t_{2}}-v_{t_{1}}\approx -\tilde{g}\left(t_{2}-t_{1}\right)+C_{bt_{1}}^{w}\Delta v_{t_{2}}^{t_{1}},$$

where $\Delta v_{t_{2}}^{t_{1}}=\int_{t_{1}}^{t_{2}}C_{b}^{bt_{1}}\left(t\right)y_{a}dt.$

The right hand side of (27) cannot be computed since the rotation matrix $C_{bt_{1}}^{w}$ is unknown without initial attitude. However, we can derive a necessary condition that ${\bar{n}}_{v,diff}=0$ using the fact $C_{bt_{1}}^{w}$ is a rotation matrix. If ${\bar{n}}_{v,diff}=0$, norms of $\tilde{g}\left(t_{2}-t_{1}\right)$ and $\Delta v_{t_{2}}^{t_{1}}$ should be the same.

Based on this observation, we define a function representing the difference between norms of $\tilde{g}\left(t_{2}-t_{1}\right)$ and $\Delta v_{t_{2}}^{t_{1}}$ as follows:

(28) $$f\left(t_{1},t_{2}\right)=||\Delta v_{t_{2}}^{t_{1}}||-g\left(t_{2}-t_{1}\right).$$

We introduce a condition to test whether ${\bar{n}}_{v,diff}\approx 0$.

**Condition** **1.** *Given acceleration and rotation rate signals between two poses $t_{1}$ and $t_{2}$, when the velocity difference signal is small, the following condition is satisfied:*

(29) $$|f(t_{1},t_{2})|<\epsilon ,$$

*where ϵ∈R is a small threshold.*

In the proposed algorithm, (24) is used as a measurement equation for estimating $q_{e}$ when the condition (29) is satisfied.

### 3.3. Practical Implementation

In a standard Kalman-based inertial navigation system, measurement detectors are used to identify the sampling instances where the measurement equations are to be applied. In this section, several heuristic detectors using for the proposed algorithm are presented, which are based on accelerometer and gyroscope outputs.

#### 3.3.1. No-Motion Detection

Gravity measurement update is utilized under the condition that no significant linear acceleration is present. An acceleration-moving-variance detector or an acceleration-magnitude detector can be used to determine when the IMU is stationary. A discrete time index *k* belongs to a non-moving interval if exist a moving window around *k* that satisfy the following conditions:

(30) $$||y_{a,i}-y_{a,i-1}||\leq \delta_{a},\;\forall k-\frac{N_{a}}{2}\leq i\leq k+\frac{N_{a}}{2}$$

where $\delta_{a}$ is threshold value and $N_{a}$ is specified window length for detecting the non-moving intervals.

Zero-angular-rate update (ZARU) measurement is performed when no remarkable rotation is present. This can be determined by using a magnitude detector as follows:

(31) $$||y_{g,i}||\leq \delta_{g},\;\forall k-\frac{N_{g}}{2}\leq i\leq k+\frac{N_{g}}{2}$$

where $\delta_{g}$ is threshold value and $N_{g}$ is specified window length.

A combination of above-mentioned detectors can be used to detect the zero-velocity intervals. This paper mainly focuses on the attitude estimation problem in a continuous walking scenario with inertial sensor attached on human upper body other than on one’s feet. The foot-mounted sensor attitude estimation result can be improved by applying ZUPT during each stance phase of the gait cycle. However, when a sensor is mounted on human body, zero-velocity intervals are only detected at the stand still periods just before and after the walking trial.

#### 3.3.2. Step Detection

In order to apply velocity-aided measurement update, walking step indices are required. In this paper, a step event is detected from accelerometer output signal. Due to the walking model of a waist-mounted IMU [28], the lowest position of the waist in a gait cycle corresponds to the high peak of the acceleration norm. Therefore, two consecutive detected high peaks of the acceleration norm data can be used to determine a walking step (see Figure 1), and each high peak also corresponds to a double stance instant.

Before applying the peak detection algorithm, the acceleration norm data is filtered using a zero-phase low-pass filter with a normalized cutoff frequency of 0.2π radians/sample. The step event is then determined with the local high peaks using Algorithm 1.
**Algorithm 1** Step Detection Algorithm.    **Input**: Filtered acceleration norm ${\tilde{y}}_{a}$, threshold value $B_{th}$, window length $N_{s}$.    **Output**: Double stance indices $P_{H}$ at high peak of acceleration norm.1:Compute the number of samples *N* of ${\tilde{y}}_{a}$2:**for** discrete time *k* from $k=N_{s}+1$ to $N-N_{s}$ **do**3: **if** ${\tilde{y}}_{a,k}>B_{th}$ and ${\tilde{y}}_{a,k}\geq \max\left({\tilde{y}}_{a,k-N_{s}}:{\tilde{y}}_{a,k-1}\right)$ and ${\tilde{y}}_{a,k}\geq \max\left({\tilde{y}}_{a,k+1}:{\tilde{y}}_{a,k+N_{s}}\right)$ **then**4:  $P_{H}=\left[P_{H},k\right];$5: **end if**6:**end for**

#### 3.3.3. Proposed Kalman-Based Filter with Velocity Observation Measurement Update

**Figure 2:**
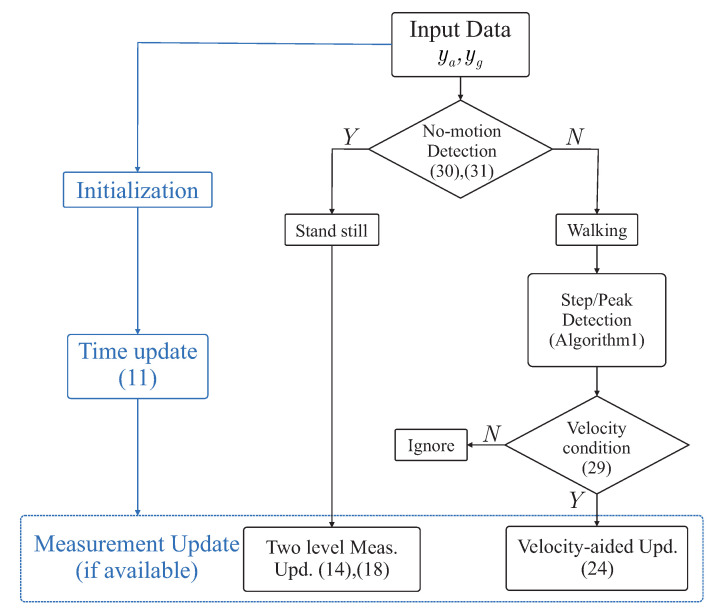
Proposed Kalman filter based attitude estimation algorithm.

The proposed Indirect Kalman-based filter is summarized in the following Figure 2 and Algorithm 2.
**Algorithm 2** Proposed Kalman-based filter with velocity observation measurement update.1:System Initialization.2:Load Initial Values.3:Compute State transition matrix.4:Compute Prior state $x_{k}^{-}$.5:Compute Prior error covariance.6:Integrate quaternion $q_{k}$.7:**if** (30) is satisfied (GM available) **then**8: Calculate the residual (17).9: **if** (31) is satisfied (ZARU update available) **then**10:  Update the residual (21).11: **end if**12: Calculate Kalman gain, Update Error covariance.13: Calculate state $x_{k}$.14: Update quaternion $q_{k}$ with (6).15:**end if**16:**if**$k\in P_{H}$ (Step is detected) **then**17: **if** (29) is satisfied (${\bar{n}}_{v,diff}\approx 0$) **then**18:  Calculate the residual (24).19:  Calculate Kalman gain, Update Error covariance.20:  Calculate state $x_{k}$.21:  Update quaternion $q_{k}$ with (6).22: **end if**23:**end if**24:Goto 3.

## 4. Experiment and Results

### 4.1. Implementation System

This paper uses a consumer-grade IMU MTi-1 sensor from Xsens Technology B.V. where the parameters are listed in Table 1.

**Figure 3:**
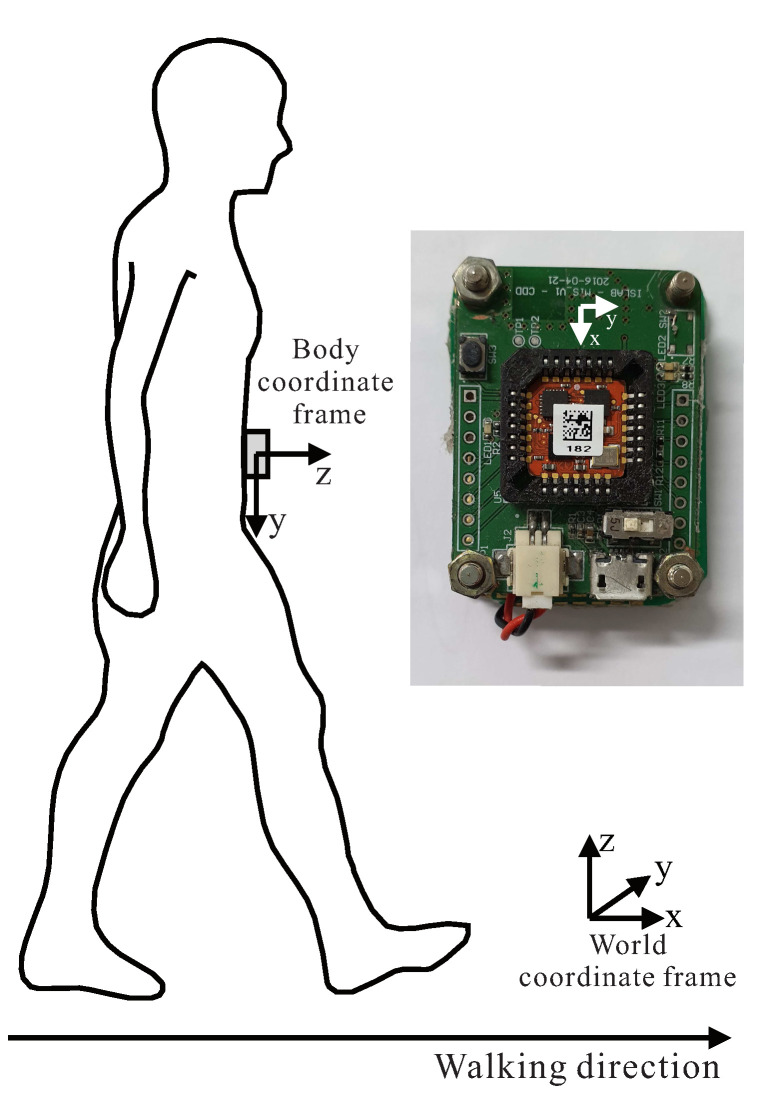
Implementation system with registered coordinate system.

The wearable node has a size of 4 cm × 3 cm × 1 cm (length × width × height) and consists of an Xsens MTi-1 sensor, a micro SD card slot and a Nordic nRF51822 micro-controller with Bluetooth Low Energy (BLE) supported. The node is attached to different user body locations using a velcro tape. Inertial sensor data were sampled at 100 Hz and saved to the SD card for post-processing. An implementation system is given with registered coordinate system in Figure 3.

### 4.2. Experiment Description

In order to verify the accuracy of the proposed algorithm, five healthy persons were recruited with information given in Table 2.

Three experiments were conducted to evaluate the performance of the proposed algorithm. The first experiment was performed inside laboratory space with the help of a motion capture system consisting of six infrared cameras. A rigid frame with three infrared markers was mounted in place with the sensor module. The module was then attached to user’s waist. Participants were asked to walk five times in a 3-to-5-m straight line inside working range of the motion capture system. Position data from the tracking system were used for detecting gait cycle and generating reference value for attitude estimation. However, to install a motion capture system, a wide area and a large number of infrared cameras were required. Therefore, equipping a motion capture system for a long walking distance experiment was impractical.

The second experiment was to verify the estimated attitude accuracy during medium walking distance. Volunteers were asked to alternately attach the sensor unit on their waist, neck, and wrist. A corridor 15-m-length was used to take the sensor recording. Each volunteer walked five times along the corridor at their preferred speed.

In the last experiment, users were asked to walk freely two times in long distance (75-m-long in particular), and with varying speed during the trial. The sensor output in all experiments was sampled at frequency of 100 Hz.

### 4.3. Estimation Results

**Figure 4:**
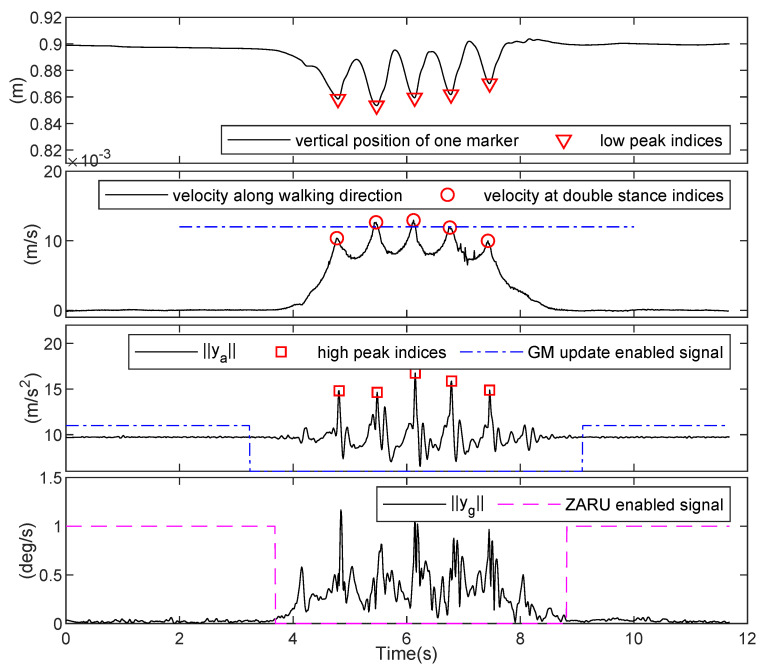
Top: vertical position of one marker from motion capture system; second: velocity along walking direction from motion capture system; third: acceleration norm, detected high peak indices, and Gravity Measurement (GM) update enabled signal; bottom: gyroscope output norm, and Zero Angular Rate Update (ZARU) enabled signal.

Position data from motion capture system in the first experiment enabled us to obtain gait parameters. Since the sensor and markers module were mounted on user waist, the vertical position of a marker represented the displacement of user’s center of mass. Therefore, double stance (DS) indices were equivalent to low peak indices of the vertical position. It can be seen from Figure 4 that double stance indices were also coincident with the high peak indices of the acceleration norm signal. The velocity signal along walking direction also supported our statement in Section 3.2 that during middle of the walking trial, the difference of the velocity at double stance instant of two consecutive strides was relatively small.

**Figure 5:**
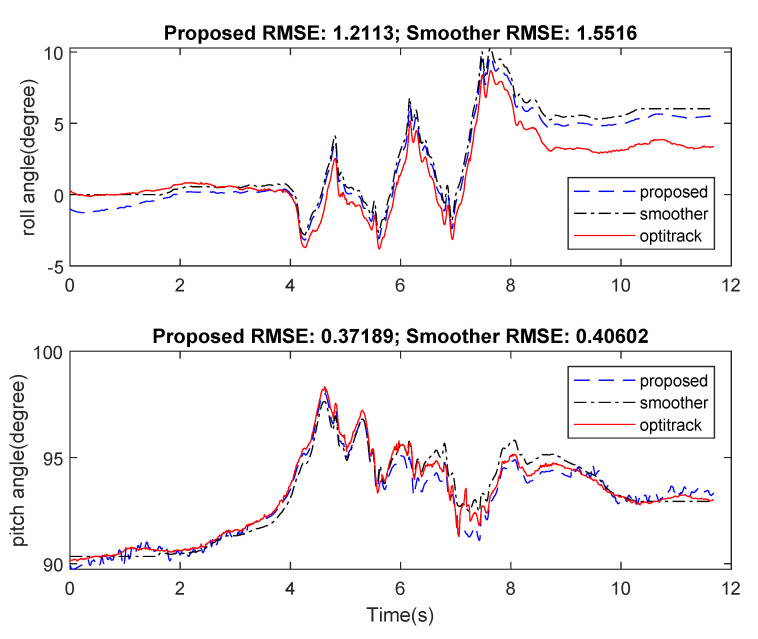
Estimated pitch and roll from smoother and proposed method in comparison with ground truth from motion tracker system.

**Figure 6:**
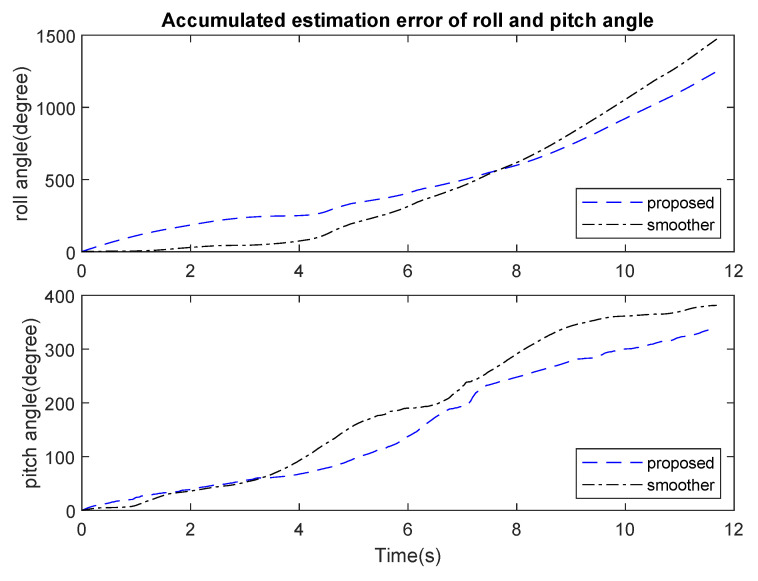
Accumulated error of roll and pitch angle from smoother and proposed method.

In the first experiment, estimated attitude using the proposed algorithm was compared with an offline Kalman-based smoother algorithm [19], which was formulated in the form of a quadratic optimization problem. Ground truth values were calculated from position of three markers. Figure 5 and Figure 6 show a slightly better result from the proposed method in comparison with the smoother. Both results, however, were still very small with the Root Mean Square Error (RMSE) for roll and pitch being less than 2 degrees.

In the last two experiments, ground truth from the motion tracker system was not available for long walking distance. Therefore, the smoother algorithm in experiment 1 was chosen to provide reference attitude values. The proposed algorithm was compared with two standard Kalman filters. The first filter, denoted as “KF1” was a standard Kalman filter with zero-velocity measurement update during standing still periods. The target position, velocity, and orientation are estimated. The roll and pitch angle were calculated from estimated quaternion. The second one, denoted as “KF2” was a standard Kalman filter with gravity measurement and zero angular rate update which is given in Section 3.1. Only quaternion and gyroscope bias were estimated and the roll and pitch outputs were from the estimated quaternion. Algorithm descriptions are given in Table 3.

Since the estimation error was caused by accumulating error from integration process, standard Kalman filter usually achieved good performance only at an early stage. Hence, we could evaluate the estimation performance by comparing the estimation result at some steps at the end of each trial in the experiment.

**Figure 7:**
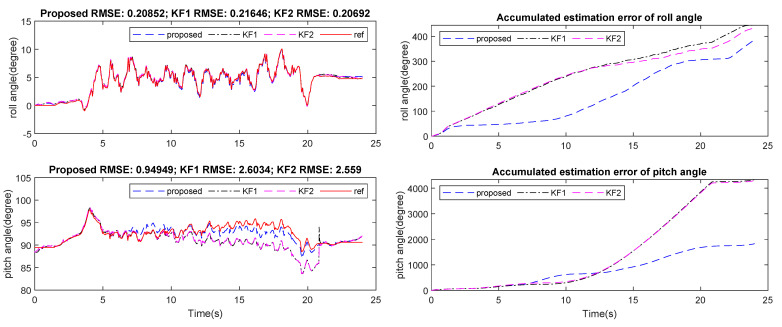
Waist-mounted roll and pitch angles estimation and errors on 15 m walking trial.

**Figure 8:**
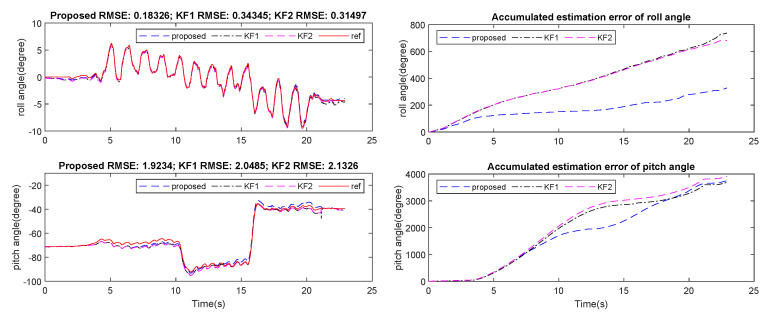
Neck-mounted roll and pitchangles estimation and errors on 15 m walking trial.

**Figure 9:**
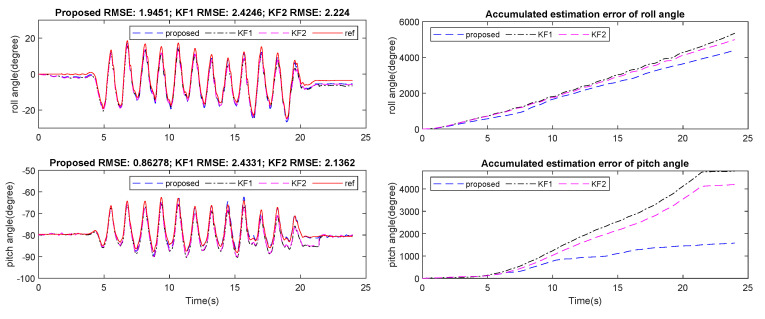
Wrist-mounted roll and pitch angles estimation and errors on 15 m walking trial.

Figure 7, Figure 8 and Figure 9 show an example of the estimated roll and pitch angle estimation and its estimation errors on a 15 m walking trial with three different sensor attachment positions. The result illustrated the similar performance of two standard Kalman filters (KF1 and KF2). The proposed algorithm gave better results in comparison with two aforementioned filters with different measurement updating methods. In particular, maximum estimation errors for both roll and pitch angles were less than 3 degrees for all sensor positions, which was approximately 50% better than two standard Kalman filters (see Table 4).

**Figure 10:**
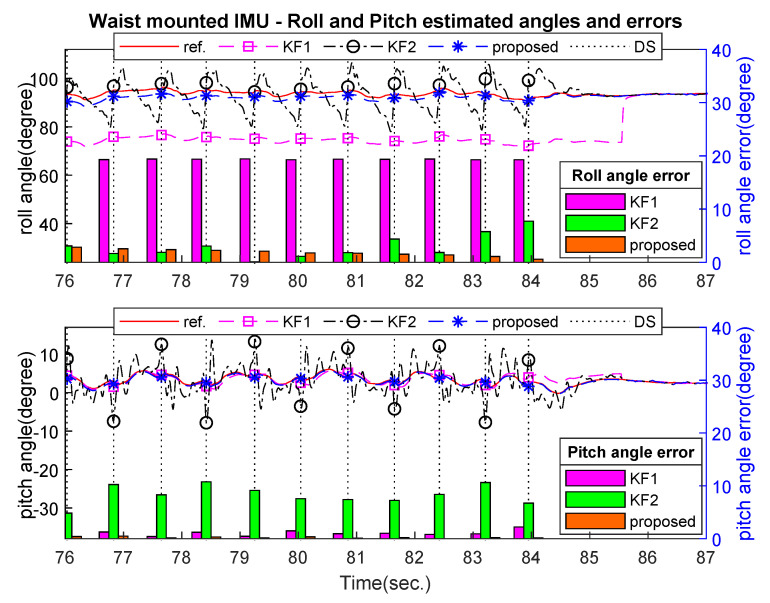
Last 10 steps of waist-mounted roll and pitch angles estimation and errors on 75 m walking trial.

**Figure 11:**
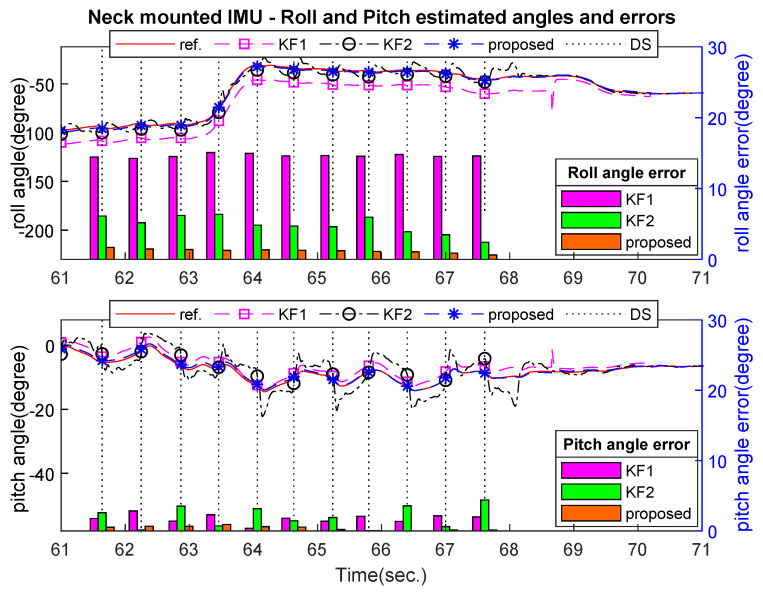
Last 10 steps of neck-mounted roll and pitch angles estimation and errors on 75 m walking trial.

**Figure 12:**
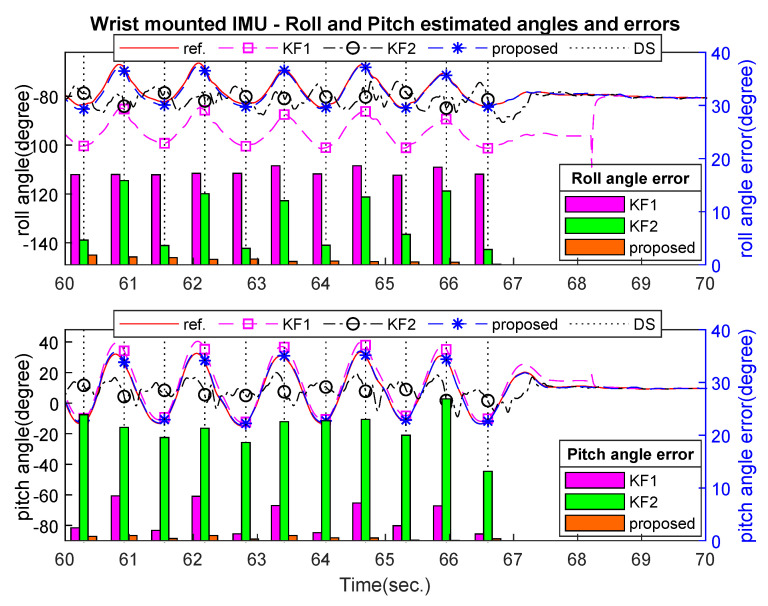
Last 10 steps of wrist-mounted roll and pitch angles estimation and errors on 75 m walking trial.

Experiment 2 was conducted for a longer walking distance and with varying walking speed. Sensors were also attached on user’s waist, neck and wrist, respectively. The roll and pitch angle estimation RMSE of the last 10 steps are presented in Figure 10, Figure 11 and Figure 12 and Table 5.

Generally, two filters KF1 and KF2 showed errors of about 15 to 20 degrees in roll angle estimation, and about 5 to 10 degrees in pitch angle error, which was 4 to 5 times worse in comparison with short walking cases. However, the proposed filter showed similar performance with the short walking distance cases, which was approximately 3 degrees maximum for both roll and pitch angles for all sensor positions.

## 5. Discussion

In this paper, a novel filter with a new measurement update has been presented for attitude estimation problem in usual human walking scenarios. The proposed algorithm was evaluated under various circumstances to investigate the variations in the estimation performance. Moreover, three different approaches, a standard Kalman filter with zero-velocity updating (KF1), a standard Kalman filter with two-level measurement observation (KF2) and an offline smoothing algorithm were discussed to compare the performance. The experiments show a promising result with estimation root mean square error smaller than 3 degree for both roll and pitch angles in all three sensor positions. The results for long walking distance indicate the robustness of the proposed algorithm with varying speed and walking distance. Based on a standard Kalman filter with the enhancing measurement update, the proposed algorithm can be used when real-time attitude estimation is necessary.

By introducing a new measurement updating method, the proposed algorithm has enhanced the attitude estimation performance. However, given the required conditions of the experiments, the sort of situations in which the proposed algorithm could be used are limited. That is, people with a lot of gait inconsistency or who have a movement disorder would be excluded in the proposed algorithm. This may open up new directions for our future works, where abnormal gait analysis is required. More complex scenario would be added in future works, such as changing direction and altitude while walking, as well as the presence of obstacles and unforeseen behaviors.

## Figures and Tables

**Table 1:** Xsens MTi-1 sensor parameters.

IMU	Accelerometer	Gyroscope
Axes	3	3
Std. Full range	±16 [g]	±2000 [${}^{\circ }$/s]
Noise density	200 [g/*√*Hz]	0.01 [${}^{\circ }$/s/*√*Hz]
Sampling Freq.	100 Hz	100 Hz

**Table 2:** The five subjects’ information.

Volunteer	Age	Weight (kg)	Height (cm)
Range	25–33	53–72	160–182
Mean	28.6	66.7	169.6
Standard deviation	3.05	6.92	5.24

**Table 3:** Algorithm descriptions.

Algorithm	Usage	Method
KF1	Experiment 2,3	Kalman filter with ZUPT
KF2	Experiment 2,3	Kalman filter with GM and ZARU
Proposed	Experiment 1,2,3	Kalman filter with GM, ZARU, and
		velocity observation measurement update
Smoother	Experiment 1,2,3	Kalman based smoothing algorithm
		with quadratic optimization

**Table 4:** Second experiment: last 10 steps of 15 m walking trials estimation RMSE.

Mounted Sensor			Maximum Error	RMSE	Standard Deviation
Waist	Roll	KF1	5.79	4.77	0.07
		KF2	13.45	6.09	3.45
		Proposed	3.11	1.69	0.60
	Pitch	KF1	1.02	0.36	0.11
		KF2	15.48	8.78	2.38
		Proposed	0.59	0.20	0.09
Neck	Roll	KF1	4.25	3.65	0.16
		KF2	9.63	5.42	1.52
		Proposed	2.20	1.15	0.42
	Pitch	KF1	2.11	1.49	0.22
		KF2	5.25	2.18	1.32
		Proposed	1.46	0.90	0.22
Wrist	Roll	KF1	5.70	5.12	0.16
		KF2	21.54	9.90	5.61
		Proposed	3.03	1.77	0.58
	Pitch	KF1	2.49	1.21	0.70
		KF2	30.97	20.94	6.59
		Proposed	1.58	0.57	0.37

**Table 5:** Third experiment: last 10 steps of 75 m walking trials estimation RMSE.

Mounted Sensor			Maximum Error	RMSE	Standard Deviation
Waist	Roll	KF1	20.89	20.05	0.08
		KF2	7.81	3.30	2.33
		Proposed	2.99	1.91	0.68
	Pitch	KF1	2.34	1.05	0.71
		KF2	10.78	8.51	1.38
		Proposed	0.60	0.26	0.16
Neck	Roll	KF1	15.62	14.88	0.24
		KF2	6.63	4.98	1.27
		Proposed	2.21	1.32	0.34
	Pitch	KF1	2.84	1.35	0.67
		KF2	4.52	2.27	1.57
		Proposed	0.94	0.48	0.31
Wrist	Roll	KF1	20.06	18.08	0.74
		KF2	15.86	8.31	5.02
		Proposed	1.84	0.97	0.54
	Pitch	KF1	8.50	3.90	3.21
		KF2	26.90	21.35	2.98
		Proposed	1.21	0.48	0.37

## Data Availability

The datasets used and/or analyzed during the current study are available from the corresponding author upon reasonable request.

## References

[1] Barbour N., Schmidt G. (2001). Inertial sensor technology trends. IEEE Sens. J..

[2] Hellmers H., Norrdine A., Blankenbach J., Eichhorn A. An IMU/magnetometer-based Indoor positioning system using Kalman filtering. Proceedings of the International Conference on Indoor Positioning and Indoor Navigation.

[3] Jimenez Ruiz A.R., Seco Granja F., Prieto Honorato J.C., Guevara Rosas J.I. (2012). Accurate Pedestrian Indoor Navigation by Tightly Coupling Foot-Mounted IMU and RFID Measurements. IEEE Trans. Instrum. Meas..

[4] Kinsey J.C., Eustice R.M., Whitcomb L.L. Survey of underwater vehicle navigation: Recent advances and new challenges. Proceedings of the Conference on Manoeuvring and Control of Marine Craft.

[5] Godha S., Cannon M.E. Integration of DGPS with a Low Cost MEMS-Based Inertial Measurement Unit (IMU) for Land Vehicle Navigation Application. Proceedings of the 18th International Technical Meeting of the Satellite Division of the Institute of Navigation (ION GNSS 2005).

[6] Demrozi F., Pravadelli G., Bihorac A., Rashidi P. (2020). Human Activity Recognition Using Inertial, Physiological and Environmental Sensors: A Comprehensive Survey. IEEE Access.

[7] Sousa Lima W., Souto E., El-Khatib K., Jalali R., Gama J. (2019). Human activity recognition using inertial sensors in a smartphone: An overview. Sensors.

[8] Bennett T.R., Wu J., Kehtarnavaz N., Jafari R. (2016). Inertial measurement unit-based wearable computers for assisted living applications: A signal processing perspective. IEEE Signal Process. Mag..

[9] Yang D., Huang J., Tu X., Ding G., Shen T., Xiao X. (2019). A wearable activity recognition device using air-pressure and IMU sensors. IEEE Access.

[10] Loianno G., Brunner C., McGrath G., Kumar V. (2017). Estimation, control, and planning for aggressive flight with a small quadrotor with a single camera and IMU. IEEE Robot. Autom. Lett..

[11] Alatise M.B., Hancke G.P. (2017). Pose Estimation of a Mobile Robot Based on Fusion of IMU Data and Vision Data Using an Extended Kalman Filter. Sensors.

[12] Suh Y.S. (2010). Orientation Estimation Using a Quaternion-Based Indirect Kalman Filter With Adaptive Estimation of External Acceleration. IEEE Trans. Instrum. Meas..

[13] Kok M., Hol J.D., Schon T.B. (2017). Using Inertial Sensors for Position and Orientation Estimation. Found. Trends Signal Process..

[14] Roetenberg D., Luinge H.J., Baten C.T.M., Veltink P.H. (2005). Compensation of magnetic disturbances improves inertial and magnetic sensing of human body segment orientation. IEEE Trans. Neural Syst. Rehabil. Eng..

[15] Ryu J., Gerdes J.C. (2004). Integrating Inertial Sensors with Global Positioning System (GPS) for Vehicle Dynamics Control. ASME J. Dyn. Syst. Meas. Control.

[16] Weinberg M.S., Wall C., Robertsson J., O’Neil E., Sienko K., Fields R. (2006). Tilt Determination in MEMS Inertial Vestibular Prosthesis. ASME J. Biomech. Eng..

[17] Seel T., Raisch J., Schauer T. (2014). IMU-Based Joint Angle Measurement for Gait Analysis. Sensors.

[18] Jarchi D., Pope J., Lee T.K.M., Tamjidi L., Mirzaei A., Sanei S. (2018). A Review on Accelerometry-Based Gait Analysis and Emerging Clinical Applications. IEEE Rev. Biomed. Eng..

[19] Suh Y.S. (2014). Inertial sensor-based smoother for gait analysis. Sensors.

[20] Rehbinder H., Hu X. Drift-free attitude estimation for accelerated rigid bodies. Proceedings of the 2001 ICRA. IEEE International Conference on Robotics and Automation.

[21] Muro-de-la-Herran A., Garcia-Zapirain B., Mendez-Zorrilla A. (2014). Gait analysis methods: An overview of wearable and non-wearable systems, highlighting clinical applications. Sensors.

[22] Tao W., Liu T., Zheng R., Feng H. (2012). Gait analysis using wearable sensors. Sensors.

[23] Glowinski S., Krzyzynski T., Bryndal A., Maciejewski I. (2020). A Kinematic Model of a Humanoid Lower Limb Exoskeleton with Hydraulic Actuators. Sensors.

[24] Rab G., Petuskey K., Bagley A. (2002). A method for determination of upper extremity kinematics. Gait Posture.

[25] Kavanagh J.J., Barrett R.S., Morrison S. (2004). Upper body accelerations during walking in healthy young and elderly men. Gait Posture.

[26] Ojeda L.V., Adamczyk P.G., Rebula J.R., Nyquist L.V., Strasburg D.M., Alexander N.B. (2019). Reconstruction of body motion during self-reported losses of balance in community-dwelling older adults. Med. Eng. Phys..

[27] Lupton T., Sukkarieh S. (2012). Visual-Inertial-Aided Navigation for High-Dynamic Motion in Built Environments Without Initial Conditions. IEEE Trans. Robot..

[28] Do T., Liu R., Yuen C., Zhang M., Tan U. (2016). Personal Dead Reckoning Using IMU Mounted on Upper Torso and Inverted Pendulum Model. IEEE Sens. J..

